# Protection of Dairy Cattle in the EU: State of Play and Directions for Policymaking from a Legal and Animal Advocacy Perspective

**DOI:** 10.3390/ani9121066

**Published:** 2019-12-02

**Authors:** Elena Nalon, Peter Stevenson

**Affiliations:** 1 Eurogroup for Animals, rue Ducale 29, 1000 Brussels, Belgium; 2 Compassion in World Farming, River Court, Mill Lane, Godalming GU7 1EZ, UK

**Keywords:** dairy cows, animal welfare, policy, European Union, legislation, good practice, rural development, lameness, mastitis, antimicrobial use

## Abstract

**Simple Summary:**

This paper presents the regulatory instruments applicable to the protection of dairy cattle in the EU and summarizes the available evidence, pointing to some serious shortcomings in enforcing the law. Corrective measures are proposed from a legal and animal advocacy perspective, with additional considerations on best practice and directions for future developments in legislation and good practice.

**Abstract:**

With the exception of a detailed Directive for calves, the welfare of dairy cattle is not regulated by species-specific legislation in the European Union. Their basic protection falls under the provisions of Directive 98/58/EC, also known as the “General Farm Animals Directive”. Article 3 of this Directive states: “Member States shall make provision to ensure that the owners or keepers take all reasonable steps to ensure the welfare of animals under their care and to ensure that those animals are not caused any unnecessary pain, suffering or injury”. However, recent reports show that the welfare of dairy cows in the EU is not sufficiently monitored and that serious problems persist. Lameness, mastitis, cubicle design, flooring, cleanliness, and permanent tethering remain critical areas. We argue that, to demonstrate compliance with Article 3 of Directive 98/58, farmers and Member States should urgently address these issues. The increasing proportion of cows that are never allowed to graze and high milk yields are also reasons for concern and will need to be addressed as a matter of priority in future EU guides to good practice and, eventually, legislation.

## 1. Introduction

European Union (EU) farmers keep around 23 million dairy cows [1] on 1.7 million farms [2] with an average milk yield per dairy cow of 7000 liters per year [3]. In 2017, the EU dairy sector produced 155 million tonnes of cows’ milk [3], and milk is the EU’s number one single agricultural product sector in terms of value, at approximately 15% of overall agricultural output [2]. Similarly to what is happening in other parts of the world, where dairy farming is already highly industrialized [4], the EU dairy sector is undergoing profound changes, with fewer farms managing increasingly bigger herds and a marked predominance of the Holstein-Friesian breed [2]. At the same time, access to pasture is often not granted, in spite of its recognized benefits for cow health and welfare [5]. Farms are modernizing, with evolving technology now being capable of providing farmers with a constant flow of information concerning animal health and welfare parameters [4]. However, not much is known of the impact that these changes are having on dairy cow welfare across the EU. It is often stated that the EU has the strictest animal welfare laws in the world [6]. Indeed, the welfare of several farmed species—pigs, calves, laying hens and meat chickens—is covered by detailed species-specific EU Directives. However, there are no such welfare Directives for many other farmed species, including dairy cows. This is reason for concern, especially in the light of the outcomes of the report [7] on animal welfare prepared for the European Parliament’s Petitions Committee by the Parliament’s Directorate-General for Internal Policies stating that “dairy cow welfare […] may be considered to be the second greatest animal welfare problem in the EU”. 

The sections that follow introduce the regulatory instruments applicable to the dairy sector and summarize the evidence pointing to their inadequacy when it comes to ensuring the welfare of dairy cows. Corrective measures are proposed from a legal and animal advocacy perspective, with additional considerations on future directions for best practice and legislation. 

## 2. EU Legislation and Other International Standards on the Welfare of Dairy Cows

### 2.1. Directive 98/58/EC

In the EU, the welfare of dairy cows is covered only by Council Directive 98/58/EC concerning the protection of animals kept for farming purposes, often referred to as ‘the General Farm Animals Directive’ [8]. This Directive "lays down minimum standards for the protection of animals bred or kept for farming purposes” (Art.1). Article 3 sets out the Directive’s core principle. Specifically, it provides that “Member States shall make provision to ensure that the owners or keepers take all reasonable steps to ensure the welfare of animals under their care and to ensure that those animals are not caused any unnecessary pain, suffering or injury”. Article 3 of Directive 98/58 is couched in broad terms, but its language is strong. Its wording places legal duties on Member States and, in turn, on the owners and keepers of dairy cows (and indeed any other farmed animal species). It requires farmers to “take all reasonable steps”, to “ensure” the welfare of their animals, and to “ensure” they are not caused any unnecessary (i) pain, (ii) suffering or (iii) injury. 

### 2.2. Council of Europe Recommendation Concerning Cattle Adopted by the Standing Committee of the European Convention for the Protection of Animals Kept for Farming Purposes (the ‘Recommendation’)

Another important source concerning the welfare of dairy cows is the Council of Europe (CoE) Recommendation concerning cattle, adopted by the Standing Committee of the European Convention [9]. The European Commission’s website states: “the Council of Europe Convention on the protection of animals kept for farming purposes and its recommendations have been ratified by the EU and thus form part of Union law. Those which concern cattle must therefore also be observed” [10]. The European Commission has also said that the provisions laid down in the CoE recommendation concerning cattle must “be applied on dairy holdings within the EU” [11].

It has been argued that the CoE Recommendations made under the European Convention are only a binding part of EU law where they use the word “shall” (rather than “should”) [12]. However, even the Recommendations that use “should” are relevant in that they help interpret Article 3 of Directive 98/58. In our view, farmers who ignore a Recommendation that uses “should” may find it difficult to demonstrate that they have taken all reasonable steps to ensure the welfare of cows under their care or to ensure that they are not caused unnecessary pain, suffering or injury.

The CoE Recommendation includes provisions for all categories of cattle (heifers, dairy cows, breeding and fattening bulls, and calves). 

Key provisions that, if respected, could lead to substantial welfare benefits in the EU dairy sector, include the following:

- The need for knowledgeable stockpersons and daily inspections;
- Animals and the housing should be kept clean;
- Floors must be well drained and not be slippery;
- A lying area should be available, which consists of a solid floor covered by straw or other suitable bedding, in order to ensure comfort and to reduce the risk of injuries;
- Cubicles should allow for the species-specific movements of the animal when it stands up and lies down;
- The number of animals housed should not exceed the number of cubicles available, and it is advisable that spare cubicles should be available;
- Animals should be allowed to go outside, in the summer preferably every day;
- Painful procedures such as dehorning must be carried out under anesthesia and by a veterinarian or other person qualified under domestic legislation.

### 2.3. The OIE Standards on the Welfare of Dairy Cows

In 2015, the World Organisation for Animal Health (OIE) adopted standards on the welfare of dairy cows [13]. These are not binding. However, as with the Council of Europe Recommendation, farmers who do not respect the OIE standards may find it difficult to establish that they have taken “all reasonable steps” to ensure the welfare of their cows. Moreover, all the EU Member States are members of the OIE and accordingly, having agreed to the dairy standards, they should seek to give effect to them in their dairy sectors. The provisions of the OIE standards for dairy cattle that, if adhered to, would be particularly helpful to improve animal welfare include the following:

- Housing conditions must be clean to ensure good hygiene, comfort and minimize risk of diseases and injuries;
- Cattle need a well-drained and comfortable place to rest;
- Bedding should be provided to all animals housed on concrete;
- Cubicles should enable cows to stand and lie comfortably (e.g., length, width and height should be appropriate for the size of the largest animal). There should be sufficient room for the animal to rest and to rise adopting normal postures, and to move its head freely as it stands up;
- Where access to an outdoor area, including pasture, is possible, there may be additional benefits to dairy cattle from the opportunity to graze and exercise, especially a decreased risk of lameness.

## 3. The Welfare of Dairy Cattle in the EU: State of Play 

In the absence of a species-specific Directive, official controls on dairy cattle welfare occur in the framework of Directive 98/58. According to the European Commission, less than 1% of all EU agricultural holdings are checked every year by the competent authorities of Member States [2]. Of these, dairy and beef cattle farms constitute only a fraction. The controls are carried out to verify cross-compliance with many requirements that are relevant for payments under the Common Agricultural Policy. Therefore, animal welfare is only one among several other legal parameters that the competent authorities must check [2]. The available evidence suggests that dairy cattle welfare is not sufficiently monitored and that severe problems persist [2].

### 3.1. NGO Report on Member State Enforcement of Directive 98/58/EC Applied to Dairy Holdings

To find out to what extent Member States were enforcing the legal provisions during official controls on dairy holdings, in March 2015, Compassion in World Farming and Eurogroup for Animals contacted the responsible Ministers in all 28 Member States, explaining to them the need to take effective steps to enforce Directive 98/58 as regards the welfare of dairy cows, and asking them to complete a survey [14]. Considering the limited time and resources available to Member States to carry out official controls, the survey focused on the following areas of concern for dairy cattle welfare: lameness, cubicle size and design, injuries, tethering, and access to pasture. Twelve Member States responded: Austria, Bulgaria, Czech Republic, Denmark, Estonia, Finland, Ireland, Latvia, Netherlands, Poland, Spain and UK. In addition, the Walloon Region of Belgium responded.

It was clear from the outcomes of the survey that only a few Member States are giving any detailed consideration to what is entailed in enforcing Directive 98/58, in respect of dairy cows. Deficiencies in Member States’ approach included:

- Only two, the Netherlands and Great Britain, had set a maximum permitted level of lameness, even as a guideline;
- There appeared to be insufficient awareness of the importance to welfare of cubicle length and width, cleanliness, floor quality (neither too smooth nor rough) and reducing injuries, sores and swellings;
- Tethering was widely used in some Member States, even though it arguably does not meet the requirement in Article 3 of Directive 98/58 to take all reasonable steps to ensure the animals’ welfare and to ensure that they are not caused any unnecessary suffering;
- Generally, even when lameness, cubicle size, injuries and tethering were inspected regularly, there was no scoring system or clear threshold beyond which remedial measures must be taken and no analysis was made of the proportion of non-compliances.

### 3.2. Overview Report of the European Commission on the Welfare of Dairy Cows in the EU

In 2016, the European Commission (Directorate Food Health and Analysis) published an overview report on the welfare of dairy cows in the EU [2]. The report, based on official audits carried out in five member states (Ireland, Estonia, the UK, the Netherlands and Austria) and on a literature review, confirmed that lameness, mastitis, metabolic diseases and reproductive problems are major animal health and welfare issues across dairy production systems. However, only a few parameters are measured by the competent authorities, milk quality schemes and/or autonomously by farmers. Additionally, Member States differ in the way they record and report information on dairy farms. The most frequently recorded factors are lameness, somatic cell count, ease of calving, and body condition score. 

The report tried to form an overview of how Directive 98/58 is applied on EU dairy farms. However, no definitive results could be obtained. “Matters for consideration” for Member States to better enforce Directive 98/58, as concerns dairy cow welfare, were as follows: (i) drafting dedicated national action plans incorporating the use of animal based measures, (ii) collecting data in a consistent manner, and (iii) fostering cooperation and sharing of information and good practice among the different stakeholders of the dairy production chain. These objectives can be achieved with a combination of instruments, such as financial incentives, updated codes of practice, penalties and other regulatory tools. After the completion of the project on dairy cow welfare and publication of the overview report, the European Commission did not follow up the “Matters for consideration by Member States”. To the best of our knowledge, if we exclude industry-led initiatives and quality or animal welfare assurance schemes, there have been no major activities on the part of Member States to improve enforcement of the provisions of Directive 98/58 as regards dairy cows. It should be noted that, in 2019, the European Commission (SANTE F) started a project on “Quality controls and indicators for animal welfare”. One of the objectives of this project is to map the use of indicators by the Member States as part of their official controls of the legal requirements for animal welfare, which include those for dairy cows. A meeting is planned at the end of 2019 to discuss the first outcomes of this project with Member States [European Commission, personal communication]. 

### 3.3. Parliamentary Report on Animal Welfare in the EU

In 2017 the Policy Department for Citizens’ Rights and Constitutional Affairs of the European Parliament commissioned the report “Animal welfare in the European Union” upon request of the Committee on Petitions [7]. The report acknowledges the positive effects that EU animal welfare legislation—when correctly enforced—has had, not only on the animals but also on the global reputation of the EU and on trade agreements. However, importantly, it also stresses that, in some cases, legislation has been insufficient in improving animal welfare, and that too many animals are currently not protected by species-specific legislation. In view of the large body of scientific evidence available to policy makers and of increasing public concern over animal welfare, the report calls for a general animal welfare law that should address all the gaps currently existing in animal protection in the EU. One of the conclusions is that “The second worst problem in Europe now is poor welfare of dairy cows because of leg disorders, mastitis and reproductive problems”. It adds: “The proportion of cows affected by one or more of these disorders is high and the animals live with the poor welfare for a substantial part of their lives so, although the numbers of individuals are not the largest, the overall extent of the welfare problem is very great”. The report also states that “genetic selection of animals is the cause of welfare problems in many species”.

## 4. Areas of Concern to Achieve Compliance with Directive 98/58

Scientific research helps us to understand what are the welfare problems that affect dairy cows, as well as the “all reasonable steps” that must be taken to address these problems in order to “ensure” dairy cows’ welfare. Indeed, the European Commission has stressed, in the context of interpreting and applying Directive 98/58, that “the necessary scientific assessment of dairy cow welfare has been performed by the European Food Safety Authority (EFSA) on request by the Commission and these data have been published in several opinions on dairy cows” [11]. In light of this, the purpose of this section is to examine what is entailed for Member States in enforcing, and for farmers in complying with, the core provision of Directive 98/58: Article 3. 

### 4.1. Lameness

EFSA [5] identifies foot and leg disorders as the major welfare problem for dairy cows in terms of incidence and magnitude of adverse effect. The European Commission also recognizes that lameness “is widely regarded as one of the major welfare problems for dairy cows” [2]. The majority of estimates of lameness are within the range 20%–25%, with no reduction in prevalence in the last 20 years [15]. For instance, a recent study in the UK found that mean within-farm lameness prevalence was 31.6% [16]. The study further stresses that, *“worldwide estimates have estimated the mean within farm prevalence to range from 14 to 31%”*. EFSA [5] points out that, “Most lame cows are in pain and have greater difficulty in coping with their living conditions than non-lame cows because of the effects of the foot or leg disorder on walking, lying comfort, standing up and avoidance behaviour. Lame cows are more likely to become subordinate…and to develop mastitis and metabolic disease”. 

More recently, the Federation of Veterinarians of Europe (FVE) states in its 2019 position paper [17] that lameness is painful and “is one of the most pressing welfare problems for dairy cows on account of the numbers of animals affected, the duration for which they are affected and the severity of welfare impacts on individual affected animals.” According to EFSA [5], “when the prevalence of recognizable locomotor difficulties in dairy cattle is above 10%, this indicates that the prevention programme is inadequate”. The FVE further argues that, “lameness prevalence below 5% is achievable on commercial farms” [17]. The FVE calls for “all countries to set up a statutory maximum lameness rate, above which veterinary intervention is needed with a tailor-made plan to correct and improve the situation” [17]. 

The farmers’ duty under Article 3 of Directive 98/58 to take “all reasonable steps” to ensure welfare and to ensure their cows are not caused any unnecessary pain, suffering or injury (hereafter referred to as the “all reasonable steps” duty), means that they must strive to minimize lameness and to treat it rapidly when it occurs. In particular, they should take the following steps:

- Score lameness regularly: this is often referred to as ‘mobility scoring’. Ideally, this should be done monthly for early identification of subclinical lameness [17]; as a minimum, this must be done four times a year for lactating cows. A number of mobility scoring schemes are readily available for the purpose [18,19,20,21,22];
- Have a foot health program: the FVE recommends foot inspection with regular preventive trimming as necessary should be carried out at intervals not greater than six months by a skilled foot trimmer or veterinarian [17];
- Provide cows with a deep bed of straw or sand to encourage lying behaviour [16]: too much standing causes damaged hocks and hoof problems, leading to lameness;
- Adapt cubicle size and numbers to optimize cow health and welfare: in order to avoid excessive amounts of time being spent standing, cubicles should not be too small and should be designed to meet the cows’ physical health and behavioral needs; in addition, the barn should not be overcrowded [16,17];
- Treat lame cows promptly: the FVE points out that immediate treatment of lame cows by competent professionals has been demonstrated to improve recovery rates significantly [17]. It adds that analgesia must be provided without delay and for as long as necessary as part of the treatment of painful lameness-causing lesions.

### 4.2. Mastitis

Mastitis is a common, extremely painful disease caused by multiple factors [23]. It is a major source of pain for affected cows. EFSA states that mastitis remains a major challenge to the dairy industry and estimates that the incidence of clinical mastitis for the different EU Member States varies between 20%–35% cows per herd per year [15]. To comply with the “all reasonable steps” duty, farmers should strive to minimize mastitis and to treat it rapidly when it occurs. EFSA [5,24] recommends that the prevalence of mastitis should be reduced by:

- Preventing the transmission of infection from cow to cow or through the environment;
- Improving the immune system by minimizing stress factors and a nutritionally balanced feed intake;
- Identifying and culling carrier cows;
- Keeping cows and housing clean;
- Providing effective ventilation.

In addition, the FVE recommends that teats should be cleaned, disinfected and dried before milking and disinfected after milking, and that milking machines should be properly maintained [23]. Applying teat sealant and using mastitis vaccines may contribute to reducing the risk of mastitis [23]. Concerning the prevention of mastitis via routine dry cow treatment, the FVE states that “in a society which is critical of antimicrobial use in farm animals and where routine prophylactic use is no longer considered acceptable, there is a need to move toward standard use of selective dry cow treatment” [23]. It adds that antimicrobials should only be used “on the basis of clinical history of mastitis, suspicion of intra-mammary infection (by individual cell count and/or a positive bacterial milk culture) and individual cow or farm risk factors (e.g., damaged teats)”. 

Notably, the routine use of antimicrobials is now prohibited under Regulation (EU) 2019/6 on veterinary medicinal products [25], which will come into force on 28 January 2022. Article 107.1 provides that antimicrobials “shall not be applied routinely nor used to compensate for poor hygiene, inadequate animal husbandry or lack of care or to compensate for poor farm management”. Article 107.3 states that antimicrobials “shall not be used for prophylaxis other than in exceptional cases, for the administration to an individual animal or a restricted number of animals when the risk of an infection or of an infectious disease is very high and the consequences are likely to be severe”. 

In the light of Regulation (EU) 2019/6, farmers will need to improve early detection of mastitis and provide prompt, effective treatment of cows with mastitis, rather than relying on routine treatment of dry cows with antimicrobials.

### 4.3. Cubicle Size and Design

Cubicles are the most common form of housing for dairy cows in the EU. A number of welfare problems can arise in cubicles. EFSA [5] concludes that if cubicles are too narrow, movement difficulties and teat trampling may occur. The body length of cows has increased over the years [5]; older cubicles are too short for today’s large cows. This forces them to lie or stand with their back legs in the passageway [26]. Cubicle length and design influence cow comfort. Cows go through a sequence of movements for lying down and getting up, which may not be possible, or may be difficult and protracted, if the design of the cubicles is poor. In some cases, cows may collide with the housing equipment whilst lying down; this can result in injuries. EFSA recommends that cubicles “should be designed in such a way that the forward movement of the body of the cow is not thwarted when changing position from lying to standing [5].” 

If the lying area in the cubicles does not provide a suitable surface, cows can suffer sores [27]. Providing a bedding of straw or sand will help ensure cows spend sufficient amount of time lying for rest and rumination, and will improve the condition of their feet and reduce the incidence of lameness [28]. The importance of providing cows with comfortable lying areas is also stressed in the OIE standard [13] (Article 7.11.5.1e). 

### 4.4. Sores, Wounds, Injuries and Swellings

Sores, wounds, injuries and swellings must be minimized, both in the interests of animal welfare and because they can act as a gateway for infections to enter the cow. It follows that sharp edges and protrusions must be avoided, and damaged fittings must be repaired. EFSA [5] concludes that “well-managed deep straw or sand reduce injuries, such as skin lesions, as compared with a hard floor.” Farmers should provide prompt, effective treatment of sores, wounds, injuries and swellings, in order to fulfil their “all reasonable steps” duty.

### 4.5. Floors and Cleanliness

Good floor condition is vital to reduce lameness and injuries. Floors must be neither too rough (as this can lead to foot injuries) nor too smooth (as this can lead to cows slipping); additionally, they must be non-slip. EFSA [5] stresses that “standing and walking for prolonged periods on concrete floors, or floors that are wet or covered in slurry cause severe foot disorders”. The importance of providing cows with non-slip, clean flooring is also stressed in the OIE standard ([13] Article 7.11.5.1e). In order to respect Article 3 of Directive 98/58 farmers should ensure that floors are neither rough nor too smooth, and are not covered in slurry.

Cows, housing, feeding areas and floors must be kept clean and dry to reduce infection pressure. Griffiths et al. [16] argue that leaving accumulated slurry on the floor acts as a reservoir of bacteria and is associated with increased levels of digital dermatitis. In addition, cows prefer clean and dry surfaces for resting.

### 4.6. Tethering

In some Member States, many cows (including cows in organic dairy production) are tethered [2,29,30], i.e., they are tied up with a chain or strap around their neck that is fastened to a hook in the floor or a rail above them. In some cases, they are tethered like this for 24 h a day, all year round. Tethering only allows a cow to stand up, lie down and take a few steps backwards, forwards and sideways. This results in a highly restricted behavioral range and makes it impossible for cows to perform their natural behaviors of foraging, and investigation and manipulation of their environment (these are described in more detail in the below section on zero-grazing systems). Walking is a behavioral priority for cows [31] but is impeded when cows are kept for prolonged periods or permanently in tie-stalls. Limited walking time can result in poor claw health and reduced mobility [31,32]. Recent studies indicate that monotonous, stimulus-poor environments can lead to boredom in animals [33,34]. It may be that cows tethered permanently or for prolonged periods experience lack of stimulation and boredom. In some rare cases, cows cannot even lie properly as the tethers are too short to enable the cow to sleep with her head resting on the ground [35]. Sufficient lying time is important for cow welfare [36] and a large part of rumination takes place during lying [5]. 

Tie-stall systems also pose significant health challenges for the animals. Popescu et al. [29] compared cows kept in loose housing and in tie-stalls. They found that the “loose system is more advantageous when it comes to the feeding, housing and behaviour of the dairy cow”. Regarding health, this study reports that the percentage of cows with ocular discharge, increased respiratory rate, diarrhoea, mastitis and dystocia was significantly lower in loose housing systems than in tie-stalls. From a societal perspective, it is interesting to note that, in regions where tie stalls are still very frequent or the norm, such as the US and Canada, public acceptance of tethering is low [37].

It is arguably anomalous that EU legislation prohibits several close confinement systems including barren battery cages, sow stalls (except in the first four weeks after service) and veal crates but does not expressly prohibit a similarly confining system for dairy cows, i.e., tie-stalls. We take the view that the use of tie-stalls is potentially inconsistent with Article 3 of Directive 98/58 as farmers using this system are not taking “all reasonable steps” to:

- “Ensure” the cows’ welfare;
- “Ensure” they are “not caused any unnecessary pain, suffering or injury”.

## 5. Other Common Welfare Challenges for Dairy Cows Requiring Stricter Regulation

### 5.1. Zero-Grazing Systems

Many EU dairy cows are now ‘zero-grazed’, i.e., they have no or very limited access to pasture. Scientific research shows that such limited access to pasture has a detrimental impact on the health and welfare of dairy cows. In their overall scientific opinion [5], EFSA stated, in what they characterized as a high priority conclusion, that “If dairy cows are not kept on pasture for parts of the year, i.e., they are permanently on a zero-grazing system, there is an increased risk of lameness, hoof problems, teat tramp, mastitis, metritis, dystocia, ketosis, retained placenta and some bacterial infections.” A review of the literature by researchers in Northern Ireland concluded: “Regarding health, cows on pasture-based systems had lower levels of lameness, hoof pathologies, hock lesions, mastitis, uterine disease and mortality compared with cows on continuously housed systems” [38]. Pasture access also had benefits for dairy cow behaviour, in terms of grazing, improved lying/resting times and lower levels of aggression. Moreover, when given the choice between pasture and indoor housing, cows showed an overall preference for pasture, particularly at night.” Von Keyserlingk et al. [39] conducted a study to determine if pasture access is important to cows. They investigated to what extent cows will work to access pasture (by pushing on a weighted gate) and compared it to the motivation to access fresh feed. They found that cows worked at least as hard to access pasture as they did to access the fresh feed and worked hardest for outdoor access in the evening hours. They concluded: “Echoing public views on what allows for a good life for cattle, these results show that cows are highly motivated for outdoor access”.

Access to pasture is also important to enable cows to engage in their normal behaviours, which EFSA [15] identifies as including:

- Exercise which is needed for normal bone and muscle development;
- Foraging, which accounts for a large proportion (up to 80%) of the daily activity of cows kept in a semi-natural situation. EFSA states that “in the absence of an appropriate foraging environment, welfare can be poor”;
- Investigation and manipulation of their environment. Cows have a natural tendency to explore their environment and they show a fair amount of curiosity;
- Appropriate social interactions.

Cows cannot properly carry out these behaviours when they have limited access to pasture. In a high priority recommendation, EFSA [5] states that “When possible, dairy cows and heifers should be given access to well managed pasture or other suitable outdoor conditions, at least during summertime or dry weather”. Poor air quality is an additional concern in permanently housed cows. High levels of irritants such as ammonia and dust can make cows vulnerable to respiratory diseases. 

Considering the body of scientific evidence proving the benefits of seasonal/periodic access to pasture for the health and welfare of dairy cows, we argue that a clear recommendation to provide dairy cows with access to pasture during the grass growing season (except when weather or where geographical conditions do not allow this) should be incorporated in any future good practice guidance and legislation on dairy cattle. From a purely animal advocacy perspective, we take the view that dairy cattle should not be reared in geographical areas where seasonal grazing is not possible. 

Financial incentives for farmers who want to provide access to pasture can contribute to increase the proportion of cows that are allowed to graze at least seasonally. In their recent report on animal welfare spending in the EU, the European Court of Auditors stressed [6] that Member States can make funding available to farmers through Rural Development funding under the Common Agricultural Policy (CAP). Such funding can be used to “incentivize farmers to pursue higher [animal welfare] standards”. The European Commission has indicated that access to pasture is eligible to be included among the measures aimed at improving animal welfare, as it goes beyond minimum legislative requirements [40]. However, the Court of Auditors concluded in their report that the animal welfare measure is rarely used by Member States (only 1.57% of public expenditure in Rural Development according to the European Commission [40]) and that “the financial resources of the CAP could be better used to promote higher animal welfare standards” [6].

### 5.2. High Milk Yields

Many of today’s dairy cows have been bred for very high milk yields. Over the last twenty years, dairy farming has become more intensive, to increase the amount of milk produced by each cow [2]. Indeed, in the past forty years, milk production per cow has more than doubled [2]. EU dairy cows, mainly as a result of genetic selection, have an average yield of 6900 litres per annum, and the European Commission points out that this figure is continuing to increase annually [2]. Through selective breeding, dairy cows now produce six to ten times what they naturally would for a calf [2]. The highest yielding cows are now producing around 10,000 litres or more per annum; according to EFSA, some individual cows may even produce twice as much [15]. Animals bred for such high milk yields are vulnerable to poor welfare and a reduced lifespan. An additional concern is that the male calves of highly productive dairy breeds are of little commercial value and their on-farm management as well as their fate—live export, on-farm fattening, contract fattening or on-farm killing—is posing an increasingly big challenge for the dairy industry [41,42]. 

EFSA’s overall Scientific Opinion contains high priority conclusions, stressing that the long-term genetic selection for high milk yield is the major factor causing poor health and welfare in dairy cows [5]. This is due to the prolonged negative energy balance occurring in the highest producing animals, which causes excessive loss of body condition and predisposes dairy cows to health problems such as reduced fertility, and digestive, metabolic and infectious disease, especially mastitis. Highly productive animals also spend more time eating and may not be able to allocate sufficient time for other important activities, such as resting [15]. The concentrate supplementation needed to maintain high milk yields can lead to digestive problems, including excessive fermentation in the rumen and subacute ruminal acidosis, as well as liver abscesses, laminitis and high herd culling rates [43]. While these problems can, to some degree, be addressed by good management and nutrition, cows bred for high yields are at substantially increased risk of suffering from health disorders and it cannot be assumed that these can regularly be prevented by management practices. Moreover, EFSA has pointed out that the management practices needed to tackle these problems “may themselves reduce animal welfare, e.g., high-starch grain-based diets and minimal grazing” [15]. 

We take the view that the selection goals for dairy cows should increasingly focus on health and welfare related factors, such as enhanced disease resistance, reduced mastitis and ease of calving. Dairy cows should no longer be bred for excessive milk yield, as this leads to a range of health and welfare problems and often to premature culling, which is economically disadvantageous. Indeed, the use of high yielding dairy cows raises issues under paragraph 21 of the Annex to the General Farm Animals Directive [8]. This provides that: “No animal shall be kept for farming purposes unless it can reasonably be expected, on the basis of its genotype or phenotype, that it can be kept without detrimental effect on its health or welfare”. It is clear from the EFSA Scientific Opinion referred to above that the selection of cows for high milk yields predisposes them to health and welfare issues.

## 6. Conclusions

Tackling welfare problems in the EU dairy sector has to some extent been impeded by the lack of species-specific legislation on dairy cow welfare. However, Article 3 of Directive 98/58 should not be ignored simply because it is couched in broad language; it must be taken seriously. The available scientific literature and veterinary advice can help farmers and enforcement bodies to understand what must be done to respect Article 3’s “all reasonable steps” duty. This would contribute to tackling some of the core dairy welfare problems, such as high levels of lameness and mastitis, injuries, sores and lesions, dirty floors and animals, inadequate cubicles, and long-term use of tie-stalls. Indeed, a similar approach of using the science and veterinary advice should be used to understand the implications of Article 3 for all farmed species that are not protected by species-specific EU legislation.

In addition to the legislation that has previously been mentioned in this paper, attention should also be given to Article 13 of the Treaty on the Functioning of the European Union [44]. Article 13 requires the European Commission and the Member States, when formulating and implementing agriculture policy, to “pay full regard to the welfare requirements of animals”. We argue that the failure of the Commission and the Member States to effectively address the continuing high levels of lameness, mastitis, tethering and zero grazing is incompatible with their “full regard” duty under Article 13.

If we consider the welfare of dairy cattle from a One Health [45] and One Welfare [46] perspective, whereby the health and welfare of animals are deeply interconnected to the same aspects in human beings, it is clear that improving animal welfare in dairy farming can pay off also in terms of better protecting public health. An important example that we discussed is the prevention of mastitis as opposed to the routine intra-mammary administration of long-acting antibiotic preparations at the end of lactation, which will soon become more strictly regulated.

As a first step, the European Commission should produce a formal Recommendation (as they have done for the prevention of the routine tail docking of pigs, [47]) advising on the steps dairy farmers must take to prevent and treat lameness and mastitis as part of their legal duty to take all reasonable steps to ensure welfare and the avoidance of unnecessary pain, suffering, or injury. For their part, Member States should enforce existing rules more systematically and promote the uptake of practices that can improve the welfare of dairy cows. As recently stressed by the European Court of Auditors [6], Rural Development funding available under the Common Agricultural Policy is a powerful instrument that should be more widely used by Member States to encourage farmers to provide dairy cows with pasture access. Animal welfare outcomes (such as the incidence of mastitis or the prevalence of lameness) can be used as indicators of the success of such measures in improving animal welfare. By way of an example, in the German federal state of Lower Saxony a result-oriented measure for pigs was implemented using "intact tails" as the animal welfare indicator, with the requirement of 70 % animals with intact tails as condition for the payment [48].

Ultimately, species-specific legislation will be necessary to better protect the welfare of dairy cows in the EU. The authors fully agree with the Federation of Veterinarians of Europe, which, in 2019, called for “EU legislation on Dairy Cow Welfare to achieve minimum standards for dairy cows across the European Union, similar to the species-specific EU legislation protecting poultry and pigs” [17].

Notwithstanding the fundamental role of legislation, food businesses, including retailers, foodservice operators and food manufacturers, can also respond to societal concerns and drive change by ensuring that the farmers who supply their milk and dairy products operate to high welfare standards and, in particular, fulfill their “all reasonable steps” duty. Financial institutions such as banks should not fund dairy enterprises that do not meet this duty, while investors should be more alert in ensuring that the companies in which they invest require their suppliers of milk and dairy products to respect their Article 3 obligations. 

One last, and increasingly important, aspect to be considered is the societal acceptance of dairy farming. Recent research shows that the welfare of dairy cattle is an important issue for civil society and that some common practices, once widely known, are not easily accepted by the general public (e.g., lack of access to pasture, early cow-calf separation, and disbudding or dehorning without pain relief [49,50,51]). All this should be done keeping in mind that the concept of sustainability is evolving. According to D. Broom [52], “a system or procedure is sustainable if it is acceptable now and if its expected future effects are acceptable, in particular in relation to resource availability, consequences of functioning and *morality of action*.” It follows that if the dairy sector wants to maintain its societal licence to operate it will also need to address the expectations of the general public concerning the ethical treatment of dairy cows [53].
